# Combined microRNA and mRNA microfluidic TaqMan array cards for the diagnosis of malignancy of multiple types of pancreatico-biliary tumors in fine-needle aspiration material

**DOI:** 10.18632/oncotarget.22601

**Published:** 2017-11-21

**Authors:** Thomas M. Gress, Ludwig Lausser, Lyn-Rouven Schirra, Lisa Ortmüller, Ramona Diels, Bo Kong, Christoph W. Michalski, Thilo Hackert, Oliver Strobel, Nathalia A. Giese, Miriam Schenk, Rita T. Lawlor, Aldo Scarpa, Hans A. Kestler, Malte Buchholz

**Affiliations:** ^1^ Clinic for Gastroenterology, Endocrinology and Metabolism, University Hospital, Philipps-Universität Marburg, Marburg, Germany; ^2^ Institute of Medical Systems Biology, University of Ulm, Ulm, Germany; ^3^ Department of Surgery, Technical University of Munich, Munich, Germany; ^4^ Department of Surgery, University of Heidelberg, Heidelberg, Germany; ^5^ ARC-Net Centre for Applied Research on Cancer and Department of Pathology, University of Verona, Verona, Italy

**Keywords:** pancreatic cancer, pancreatico-biliary tumors, molecular diagnostics, fine needle aspiration biopsy, microfluidic TaqMan arrays

## Abstract

Pancreatic ductal adenocarcinoma (PDAC) continues to carry the lowest survival rates among all solid tumors. A marked resistance against available therapies, late clinical presentation and insufficient means for early diagnosis contribute to the dismal prognosis. Novel biomarkers are thus required to aid treatment decisions and improve patient outcomes.

We describe here a multi-omics molecular platform that allows for the first time to simultaneously analyze miRNA and mRNA expression patterns from minimal amounts of biopsy material on a single microfluidic TaqMan Array card. Expression profiles were generated from 113 prospectively collected fine needle aspiration biopsies (FNAB) from patients undergoing surgery for suspect masses in the pancreas. Molecular classifiers were constructed using support vector machines, and rigorously evaluated for diagnostic performance using 10×10fold cross validation. The final combined miRNA/mRNA classifier demonstrated a sensitivity of 91.7%, a specificity of 94.5%, and an overall diagnostic accuracy of 93.0% for the differentiation between PDAC and benign pancreatic masses, clearly outperfoming miRNA-only classifiers. The classification algorithm also performed very well in the diagnosis of other types of solid tumors (acinar cell carcinomas, ampullary cancer and distal bile duct carcinomas), but was less suited for the diagnostic analysis of cystic lesions.

We thus demonstrate that simultaneous analysis of miRNA and mRNA biomarkers from FNAB samples using multi-omics TaqMan Array cards is suitable to differentiate suspect solid pancreatic masses with high precision.

## INTRODUCTION

Pancreatic ductal adenocarcinoma (PDAC) exhibits the poorest prognosis of all solid tumours, with a median survival of 6 months and steadily increasing incidence rates in the industrialized world, and thus represents one of the major challenges in cancer medicine [1]. About 103,000 new cases are diagnosed each year in Europe (see data of the European Cancer Observatory at http://eco.iarc.fr/eucan/Default.aspx), and 44,000 in the USA [1]. Currently, by the time a definitive diagnosis is reached, most PDAC patients have locally advanced or metastasized disease, and are therefore not candidates for surgical resection [2]. Surgical resection of early-stage tumors is thus the only available potentially curative treatment option [2] [3], and there is an urgent need to develop early detection methods to increase the fraction of patients diagnosed in a curative stage to improve the overall outcomes of PDAC patients. Furthermore, although PDAC accounts for the majority of all malignant tumors in the pancreas [4], other neoplasms of the pancreatico-biliary system that display a wide range of biological behaviors and clinical outcomes are increasingly being recognized. Benign processes such as inflammatory masses developing in the course of chronic pancreatitis or autoimmune pancreatitis [5] [6] may cause the same signs and symptoms as malignant neoplasms. Both the timely detection and the accurate differential diagnosis of suspect masses in the pancreas are thus critical in order to avoid unnecessary delays in therapy decisions as well as potentially harmful over- or undertreatment of patients.

Presently, when the diagnosis of a suspect mass in the pancreas remains unclear, endoscopic ultrasonographyfine needle aspiration (EUS-FNA) is considered as the standard to confirm or exclude malignancy. In centers providing special requirements such as highly trained endoscopists proficient in EUS-guided biopsy approaches, equipment and on-site cytology will reach specificity of up to 96% and sensitivity of up to 87% as reported by recent systematic meta-analyses [7], [8]. However, specificity for the diagnosis of malignancy reported in the literature ranges between 80 and 100%, while sensitivity is much more variable with an overall low and variable negative predictive value (33-85%) [9]. Many factors can impact the diagnostic yield of EUS-FNA including the experience of both the endosonographer and cytologist, availability of on-site cytology, and the inherent limitations of the procedure to identify cytomorphologic features characteristic of well-differentiated cancer, in particular in the setting of chronic pancreatitis (CP). Sensitivity can be also compromised by technical factors such as sampling errors, insufficient cellularity, and the presence of fibrosis or blood [10].

New molecular approaches offer the promise of accuracy, straight-forward distribution, and affordability that will be needed to deliver practical screening tools. We have previously demonstrated that multiple types of pancreatico-biliary tumors comprising ampullary cancers, solid pseudopapillary tumors, adenocarcinomas of the distal bile duct, and inflammatory masses developing in chronic pancreatitis can be accurately differentiated via their mRNA expression profile obtained from biopsy specimens using cDNA arrays [11]. Furthermore, next generation transcriptome sequencing approaches have revealed the existence of PDAC subtypes that are of prognostic, and most likely therapeutic, relevance ([12], [13], [14], [15]). In addition to mRNA profiles, microRNAs (miRNAs) have evolved as promising biomarker molecules for cancer detection. miRNAs are highly regulated in cancer, are very stable in tissue, plasma, stool, and other fluids and can be quantified in very small sample sizes. They have extensively been studied for their role as diagnostic, prognostic or predictive biomarkers in pancreatic cancer which has been reviewed elsewhere [16], [17], [18], [19], [20], [21], [22]). However, RNA or miRNA molecular diagnostics have so far neither entered routine clinical applications nor have been used in combination to enhance their diagnostic power. Here, we present a quantitative real-time PCR-based multi-omics molecular platform that combines the classification potential of mRNA and miRNA expression patterns for the differentiation of malignant and benign pancreaticobiliary tumors in minimal amounts of biopsy material.

## RESULTS

### Generation of combined microRNA and mRNA biomarker microfluidic arrays

As mentioned above, we have previously reported that accurate molecular diagnostic classification of multiple pancreatico-biliary tumors by analysis of mRNA expression profiles from clinical samples is possible using custom cDNA arrays [11]. In order to transfer this diagnostic principle to a standardized and uniformly available technological platform, we established simultaneous analysis of mRNA and miRNA expression on a single TaqMan® Array microfluidic card ([23]; see Materials and Methods). mRNA markers to be included in the array were selected from our previous study [11]; 9 miRNA markers (miR-1246, miR-135b, miR-196a, miR-210, miR217, miR-155, miR-203, miR-148a, miR375) were selected from our unpublished data as well as reports from the literature (summarized in [22], [19], [17], [18], [16], [24], [25], [26], [20]). The final composition of the mixed TaqMan Array comprised 79 mRNA markers, 5 mRNA reference genes, 9 miRNA markers, and 2 small RNA reference genes (Table 1).

**Table 1:** Composition of the mixed TaqMan^®^ Array

Gene Name	Role
ACTG1	Marker
AMFR	Marker
ASNS	Marker
ATP7B	Marker
BAMBI	Marker
BCL2L1	Marker
CASP6	Marker
CDC2	Marker
CDH1	Marker
CDH3	Marker
18S-rRNA	Internal Control
CEACAM7	Marker
CLDN1	Marker
CLDN3	Marker
CLDN4	Marker
CLDN9	Marker
CLK1	Marker
CTGF	Marker
CTSC	Marker
CTSE	Marker
CYB561	Marker
DUSP3	Marker
DUSP8	Marker
E2F1	Marker
EGFR	Marker
ELA3A,ELA3B	Marker
EPCAM	Marker
F3	Marker
FGF2	Marker
GPR68	Marker
GRB10	Marker
GSTA2	Marker
HMGN2	Marker
HSF2	Marker
HSP90B1	Marker
IL6	Marker
ILKAP	Marker
INPPL1	Marker
KLRB1	Marker
KRT17	Marker
KRT7	Marker
LDHA	Marker
MAD2L1	Marker
MAP2K4	Marker
MMP2	Marker
MUC3A,MUC3B	Marker
MYC	Marker
NFATC1	Marker
PHLDA1	Marker
PPP1R1A	Marker
PPP2R5C	Marker
PTGFR	Marker
PVRL1	Marker
RAF1	Marker
RHOBTB3	Marker
SDC1	Marker
SERPIND1	Marker
SH3BP1	Marker
SLC19A1	Marker
SMARCA1	Marker
SPARC	Marker
STC1	Marker
TF	Marker
TGIF1	Marker
TJP3	Marker
TNC	Marker
TNFAIP3	Marker
TP53	Marker
USP12	Marker
VIM	Marker
ZNF91	Marker
TMPRSS4	Marker
MST1R	Marker
NT5E	Marker
RALB	Marker
TRIO	Marker
EZH2	Marker
MELK	Marker
PAK4	Marker
RRAS	Marker
RPLP0	Reference
PPIA	Reference
RPL37A	Reference
RPL30	Reference
RPS17	Reference
hsa-mir-1246	Marker
hsa-mir-135b	Marker
hsa-mir-196a	Marker
hsa-mir-210	Marker
hsa-miR-217	Marker
hsa-miR-155	Marker
hsa-miR-203	Marker
hsa-miR-148a	Marker
hsa-miR-375	Marker
RNU44	Reference
RNU48	Reference

### Evaluation of the mRNA/miRNA microfluidic array in surgical FNAB for the differentiation of ductal adenocarcinoma and chronic pancreatitis

**Figure 1:**
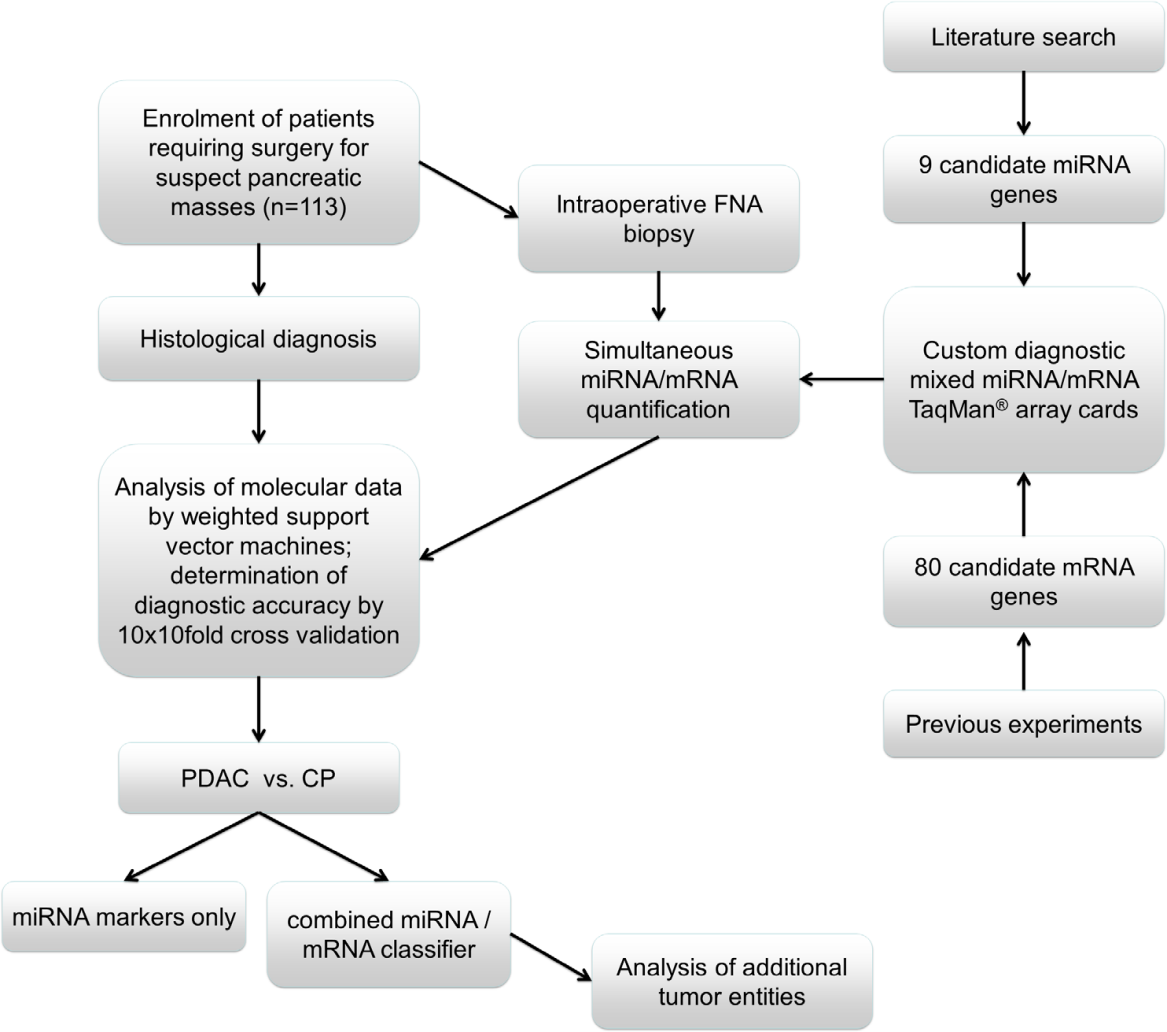
Study design

The first goal of the study was to allow an accurate molecular differentiation between PDAC and CP using fine needle aspiration biopsy samples obtained directly from tumor masses during surgery. These “surgical FNAB” (sFNAB) samples ensure both accurate targeting of the tumor mass and subsequent histological confirmation of the diagnosis in the same bioptic tissue specimen. At the same time, sFNAB closely resemble “real” FNAB samples obtained during routine diagnostic procedures in both cellular composition and overall yield of diagnostic material. A total of 113 samples from patients undergoing surgery for pancreatic masses were prospectively collected at the participating centers, including 41 from PDAC and 33 from CP patients, and subsequently subjected to gene expression analysis using the mixed TaqMan® Array (Figure 1).

**Figure 2:**
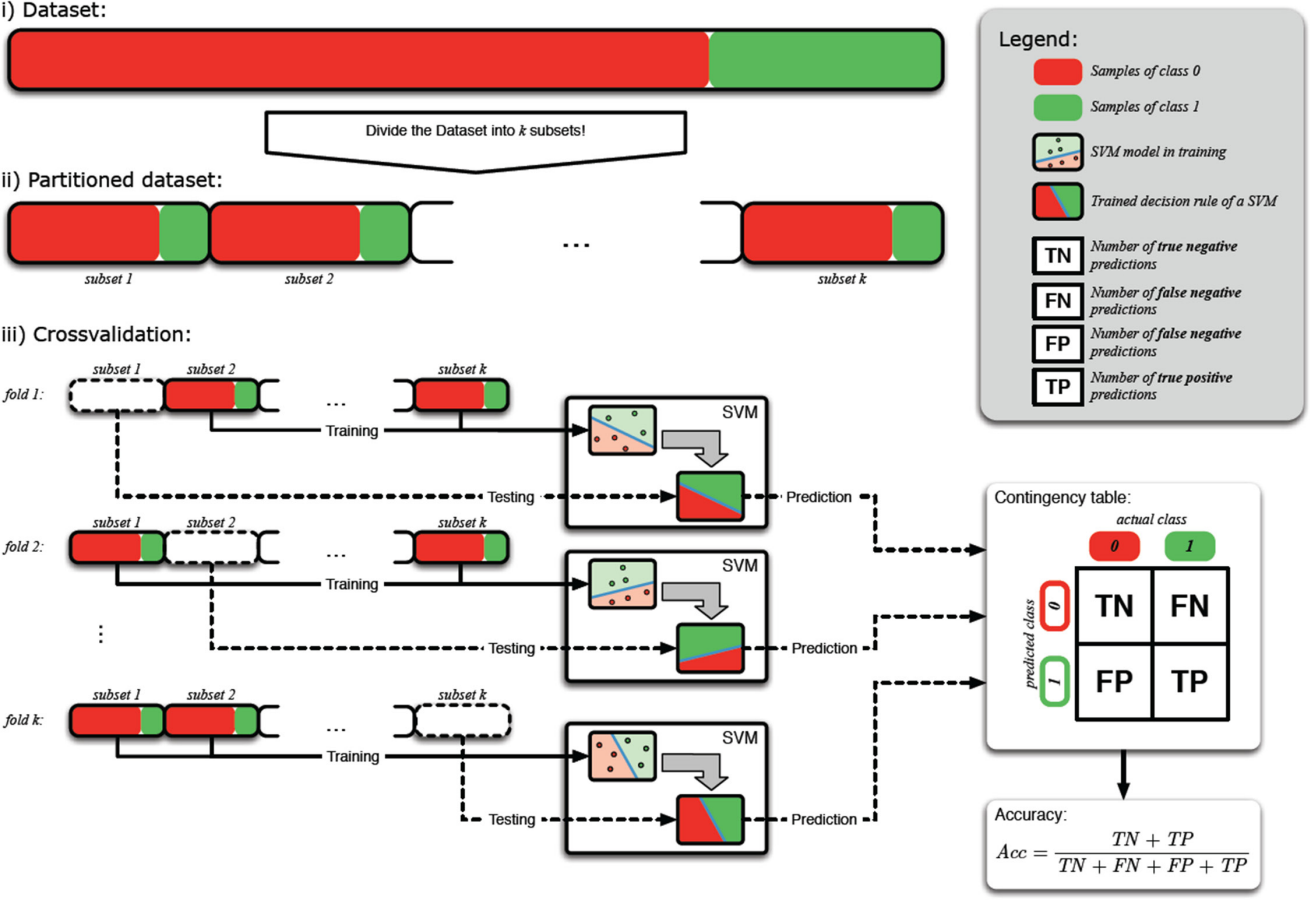
Schematic representation of 10×10 cross validation procedure The original dataset is split into ten subsets of approximately equal size. Nine of these subsets are used for training the classification model (SVM). The tenth subset is used as an independent subset for evaluating the performance of the trained classifier. This procedure is repeated for each subset and an overall accuracy is calculated as the mean accuracy over ten permutations of the original dataset.

**Figure 3:**
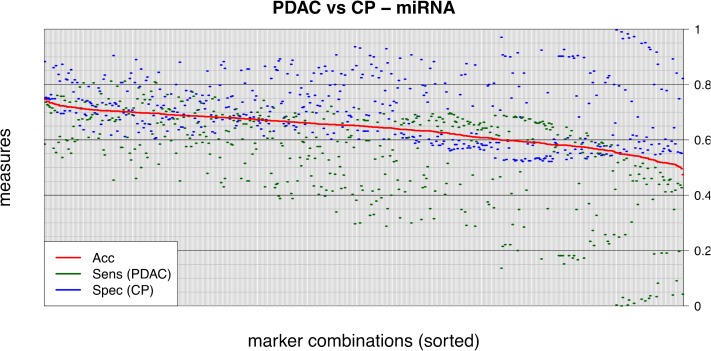
Exhaustive evaluation of all miRNA marker combinations All possible combinations of miRNA markers, from single miRNAs to the combination of all 9 markers (2^9^-1 = 511 possible combinations), were tested for their diagnostic performances on the set of PDAC and CP samples. Each combination was evaluated by a linear SVM and sensitivity, specificity and diagnostic accuracy determined by 10×10-fold cross validation. Results are plotted in decreasing order according to accuracy. Note that none of the resulting classifiers exceeded a diagnostic accuracy of 75%, with the best-performing combination achieving a sensitivity of 58.5% and a specificity of 88.3%. “CP” = chronic pancreatitis; “PDAC” = pancreatic ductal adenocarcinoma; “Acc” = accuracy; “Sens” = sensitivity; “Spec” = specificity.

In the first step of the analyses, we concentrated on the differentiation between PDAC and CP, since this is the clinically most relevant differential diagnosis. Since the selected miRNA markers had previously been reported to have high diagnostic potential on their own, we first sought to determine whether miRNA marker analysis alone would be sufficient to accurately discriminate between PDAC and CP. To this end, all possible combinations of the 9 miRNA markers (single markers, double combinations, triple combinations, and so on up to combination of all nine markers) were used to construct molecular classifiers by generating multivariate decision rules using support vector machines (SVM). Decision boundaries (= high dimensional hyperplanes) separating the two diagnostic classes were defined such that the margins between the samples of the diagnostic classes were maximized. In order to avoid overfitting of the resulting classifiers to the specific data set and to obtain an estimate of the general performance of each classifier, we performed 10×10-fold cross validation analyses. The data set was thus repeatedly randomly divided into independent training and test sets, each time training the classifier with 90% of the data and testing the diagnostic performance on the remaining 10% of samples, respectively, thereby creating a total of 100 different combinations of independent training and test sets for each candidate classifier. In the 100 independent test runs, the predicted diagnoses for all samples of the test sets were recorded and compared to their true diagnoses (using histopathological diagnosis as gold standard), resulting in a total of 740 data points for each 10×10-fold cross validation run (schematically outlined in Figure 2). Using this very stringent evaluation procedure, the best-performing classifier achieved an overall accuracy of differential diagnosis of 75% (Figure 3).

**Figure 4:**
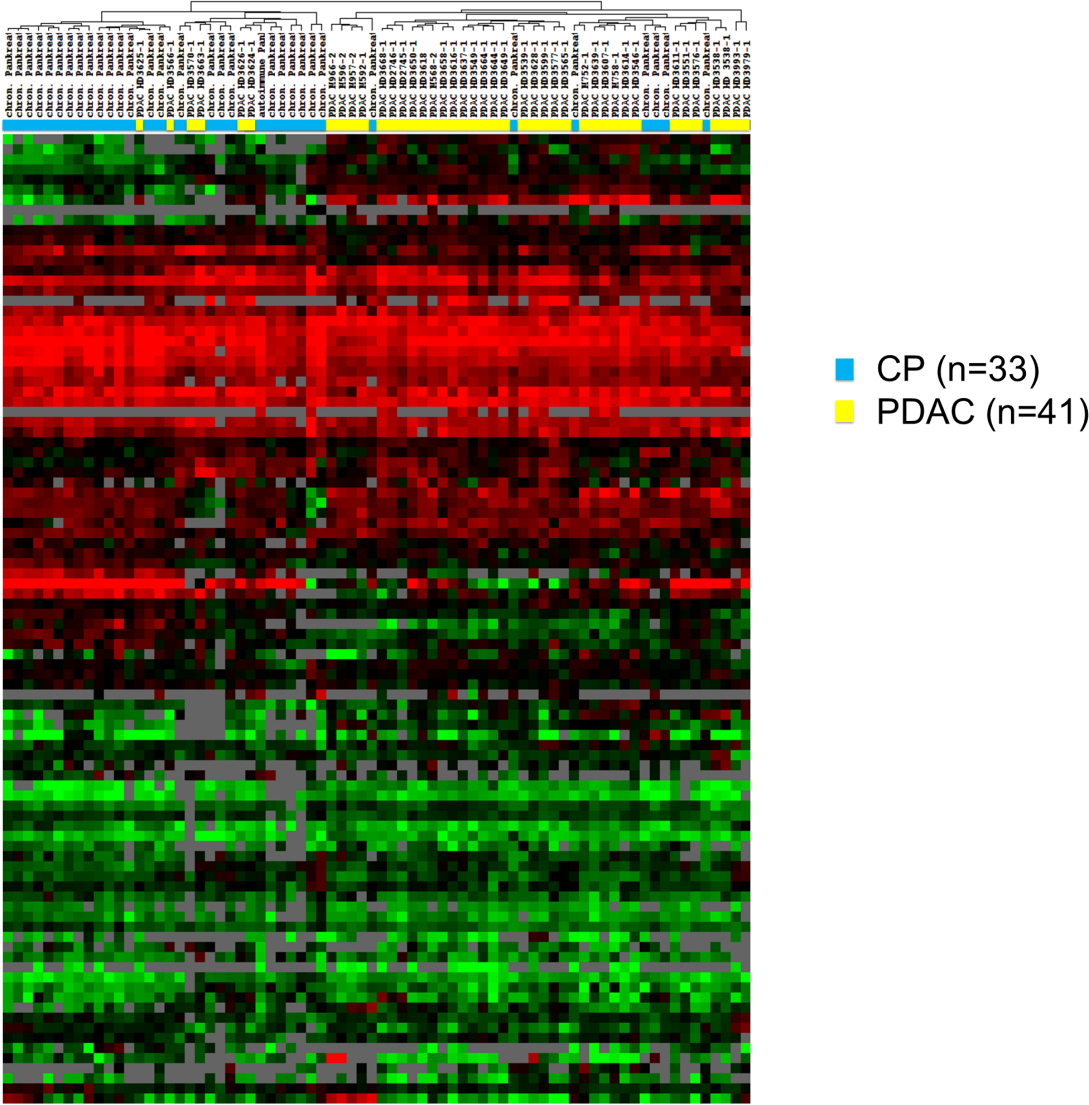
Unsupervised clustering of expression profiles from PDAC and CP biopsies Individual samples are shown in columns; genes are shown in rows. Both the samples and the genes were hierarchically clustered (complete linkage clustering) using uncentered Pearson correlation as the similarity measure. Red cells indicate high expression, black intermediate expression, and green low expression of a gene in the respective group. Clusters of CP (blue bars) and PDAC samples (yellow bars) are readily apparent. “CP” = chronic pancreatitis; “PDAC” = pancreatic ductal adenocarcinoma.

**Figure 5:**
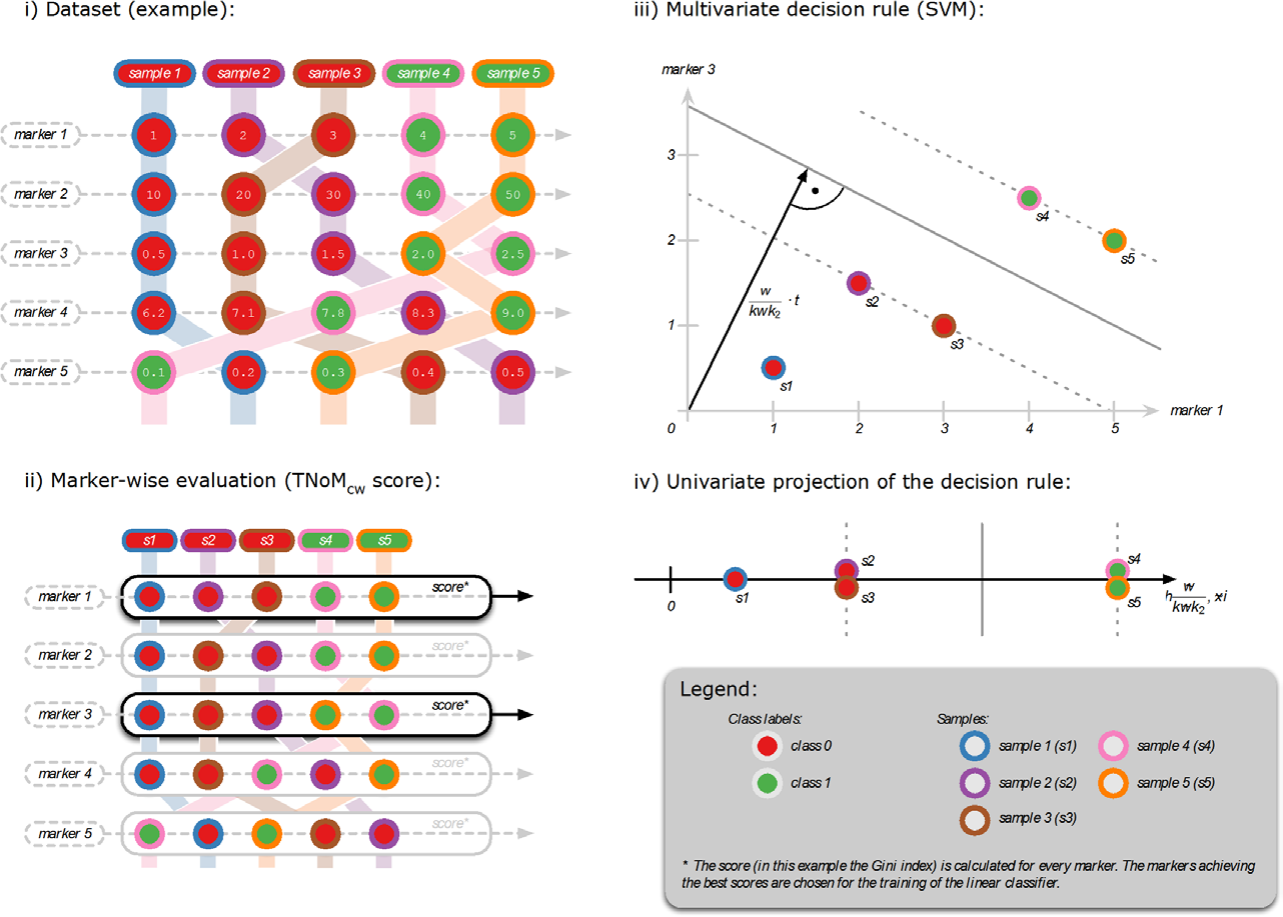
Training of mixed miRNA / mRNA classifiers Combined profiles of miRNA and mRNA markers (Panel i) were analyzed in a sequence of marker selection and classification experiment. First, a subset of candidate markers was selected via the univariate TNoM_cw_ score (Panel ii). The reduced marker profiles were then used to train a multivariate SVM (Panel iii). The final decision can be projected to univariate space (Panel iv).

**Figure 6:**
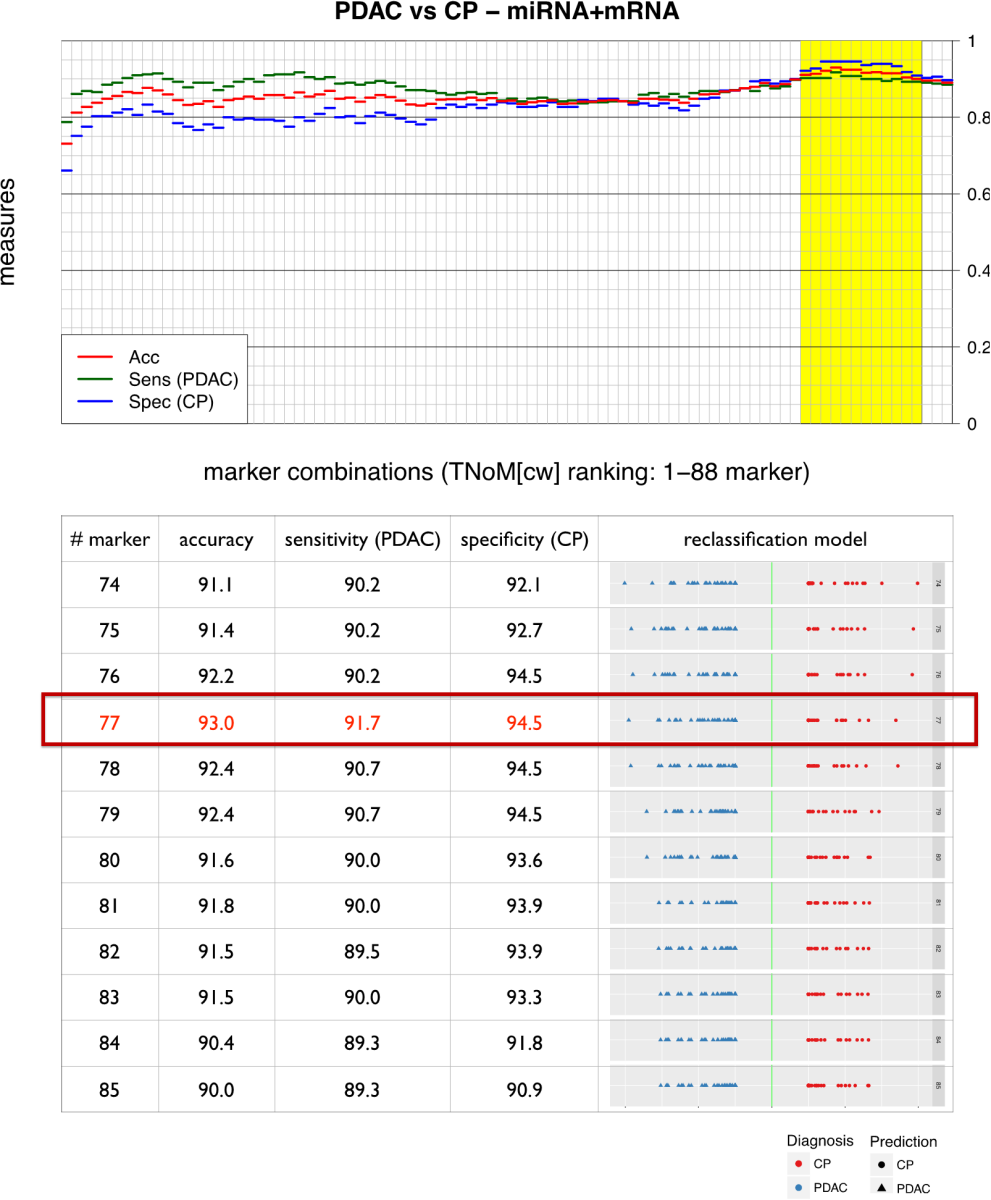
Evaluation of the combined miRNA/mRNA marker profiles Accuracy, sensitivity and specificity gained in the 10×10 cross validation experiments with the combined miRNA/mRNA marker profiles. Classification models vary in the number of markers that were selected during their training phases. Results are given in increasing order from 1 to 88 selected biomarkers. Accuracies ≥ 90% are marked in yellow. “CP” = chronic pancreatitis; “PDAC” = pancreatic ductal adenocarcinoma.

We next analysed the complete set of combined miRNA and mRNA expression data for its ability to differentiate between PDAC and CP samples. Unsupervised hierarchical clustering of the mRNA and miRNA gene expression data already showed a strong tendency for the samples to separate into two large clusters of PDAC and CP cases, with 13 out of 75 samples being placed on the “wrong” side of the cluster tree using this very simple analysis (Figure 4). In order to optimize classification, we again constructed SVM models as described above. However, exhaustive search of all possible combinations of the total set of 88 markers was not feasible due to the high number (=3,09^*^10^26^) of possible combinations. The individual markers were therefore ranked according to the sensitivity and specificity of univariate single threshold classifiers (TNoM_cw_-score), and a series of multivariate SVM classifiers was constructed by including increasing numbers of markers (1 to 88 markers, starting with the highest TNoM_cw_-score). The construction and training of classifiers is schematically outlined in Figure 5. To obtain a conservative estimate of the general performance of the classification system, we again performed 10×10-fold cross validation analyses as described above. This very rigorous evaluation procedure, which created conditions that were even more demanding than would be expected in a routine clinical scenario, revealed very good diagnostic accuracies (≥ 90.0%) for a number of classifiers comprising 74 to 85 individual markers. The highest score was recorded for a combination of 77 individual markers, with a sensitivity for the detection of PDAC of 91.7%, a specificity of 94.5%, and an overall diagnostic accuracy of 93.0% in the 10×10-fold cross validation (Figure 6). This classifier was thus chosen as the final diagnostic algorithm to be used in future clinical application.

### Evaluation of the established mRNA/miRNA classifier for identification of malignant tumors other than PDAC

**Figure 7:**
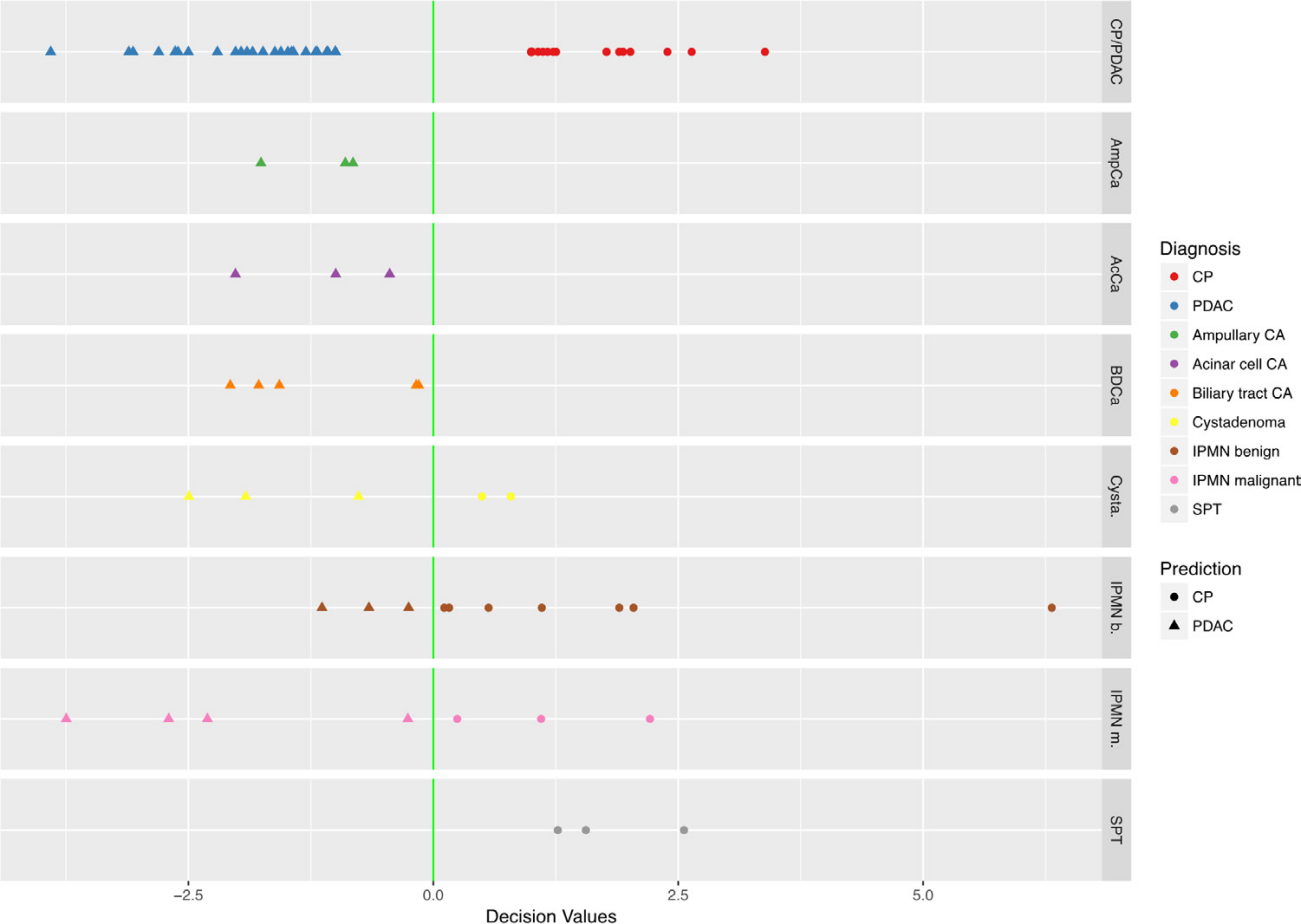
Performance of the final classifier in identifying malignancy on biopsy samples from pancreatobiliary tumors other than PDAC Retraining of the optimal model (77 markers) on all available samples. Prediction of PDAC and CP samples and categorization of all remaining tissue-types is given. “CP” = chronic pancreatitis; “PDAC” = pancreatic ductal adenocarcinoma; “CA” = carcinoma; “IPMN” = intraductal papillary mucinous neoplasm; “SPT” = solid pseudopapillary neoplasia.

During the prospective collection of samples for this study, all patients presenting with suspect masses in the pancreas were enrolled (Figure 1). A total of 39 sFNAB samples from patients for whom histopathological evaluation revealed a final diagnosis other than PDAC or chronic pancreatitis were thus also collected. The finalized diagnostic algorithm was subsequently also applied to analyze these samples. The results demonstrated that 3 of 3 ampullary carcinomas, 3 of 3 acinar cell carcinomas, and 5 of 5 biliary tract carcinomas were assigned to the “PDAC” class, thus being classified as malignant with 100% accuracy (Figure 7). In contrast, cystadenomas and IPMNs (regardless of benign or malignant status) were evenly distributed between both groups, while 3 of 3 solid pseudopapillary tumor cases were assigned to the benign (“CP”) class, indicating that the marker combination and diagnostic algorithm is less suited for the diagnostic analysis of cystic lesions.

## DISCUSSION

Currently, two major aspects are thought to contribute to the failure of diagnosis and treatment of PDAC: 1) The Genome Project has revealed that PDAC is the tumor with the highest degree of intertumoral genetic heterogeneity [14, 27], suggesting that no single molecular biomarker and no single molecular targeted therapy will work for diagnosis and treatment of all PDAC patients. 2) PDAC is an extremely stroma-rich tumor and up to 90% is made up of a highly complex assembly of activated fibroblasts, immune cells, blood vessels, neural cells and a variety of matricellular proteins [28] which contributes to therapy resistance and hinders cytological or histological diagnoses of PDAC in EUS-FNA. Accurate molecular differentiation of pancreatic cancers will thus likely require a high degree of multiplexing of markers to account for these variabilities.

At present, EUS FNA/FNAB of a pancreatic mass that is deemed resectable is not routinely performed [29], [30], [8], since a negative result cannot exclude the presence of PDAC due to the variable and low negative predictive values and will thus still lead to resection of the mass. Furthermore, there is always concern to induce tumor spreading along the biopsy channel. Tumor seeding with EUS-FNA has been reported, but the risk appears to be as small as 0,003-0,009% [31], and preoperative EUS-FNA has not been reported to be associated with adverse perioperative or long-term outcomes in patients undergoing resections for solid neoplasms of the pancreas [32]. Furthermore, neoadjuvant treatments for resectable or borderline resectable tumors or induction chemotherapies for downstaging of locally advanced non-resectable cases ([2], [33], [34], [35], [36], [37]) offer the promise of improving the outcome of PDAC patients. In such situations confirmation of the PDAC-diagnosis is mandatory before initiating neoadjuvant treatment. Thus, while currently not recommended, EUS-FNA of resectable pancreatic tumors in operable patients is likely to be invaluable for future molecular diagnosis and patient stratification if diagnostic accuracy can be substantially improved.

Multiple studies have previously been performed to evaluate the diagnostic performance of mRNA and miRNA biomarkers in PDAC. However, most of these studies were either not done in FNA material (using FFPE material instead), or did not have histopathology of all cases available to validate the biomarker results (e.g. [22], [20], [19]). We report here for the first time the simultaneous qRT-PCR-based analysis of miRNA and mRNA markers on a single microfluidic platform, interrogating FNAB samples for which detailed histopathological evaluation as gold standard is available.

### Suitability of the mRNA/miRNA multi-omics platform for the diagnosis of malignancy in various pancreatic-biliary tumors

In addition to the high sensitivity of marker detection inherent to the qRT-PCR-based technology, TaqMan Array cards are highly standardized and require limited hands-on time, making them a suitable platform for point-of-care diagnostic tests to be used in clinical routine. Our results confirm that this technology and the combined set of selected miRNA and mRNA markers is suitable to accurately distinguish between malignant and benign processes affecting the pancreas based on molecular analysis of FNAB samples. In the initial analysis of the total set of 74 PDAC and CP biopsy samples using a defined set of classifiers, we achieved a diagnostic accuracy of 100%, confirming the suitability of the multi-omics platform. The generalization ability of the support vector machine-based diagnostic algorithm, i.e., the question how accurately the algorithm would classify novel samples not previously used for training of the classifier, was addressed by performing a very rigorous 10×10-fold cross-validation procedure. This method provides a conservative estimate of the diagnostic performance that can be expected in a routine clinical setting, since 100 different combinations of independent training and test sets are interrogated, and every single misclassification event contributes to lowering of the accuracy score. In these analyses, our diagnostic system proved to be very robust, particularly in the two-class diagnostic scenario (malignant vs. benign pancreatic tumor), achieving a sensitivity of 91.7%, a specificity of 94.5%, and a diagnostic accuracy of 93.0%.

Moreover, our analyses revealed that combined classifiers comprising mRNA and miRNA markers were highly superior to classifiers based exclusively on previously reported miRNA markers. Our results for the miRNA-only classifier (75% accuracy of differentiation between PDAC and CP in 10×10fold cross validation) are in line with previous studies. Frampton and coworkers demonstrated a sensitivity of 81.5% and a specificity of 85.7% (AUC 0.930) for a 2-miRNA classifier (miR21 + miR-155) in distinguishing benign from malignant pancreatic lesions in EUS-FNAs [22]. Brand et al. reported that a 5-miRNA classifier (miR24, miR130B, miR135B, miR148A, and miR196) combined with standard cytology was able to improve the detection of PDAC to 90.8% [21]. Neither study, however, employed 10×10fold cross validation to assess the generalization ability of their classifiers.

In addition to being superior to “single omics” diagnostics in differentiating malignant from benign pancreatic masses, combining mRNA and miRNA biomarkers in a single mullti-omics molecular diagnostic platform offers additional advantages. Next generation sequencing approaches of the PDAC transcriptome have revealed prognostic and predictive subtypes that will most likely be of clinical relevance in the near future ([12], [13], [14], [15]). Most recently, Bailey et al. using NGS expression analysis defined 4 subtypes comprising: (1) squamous; (2) pancreatic progenitor; (3) immunogenic; and (4) aberrantly differentiated endocrine exocrine (ADEX) that correlate with histopathological characteristics [14]. In the same way, miRNA clusters have been associated with chemosensitivity of PDAC stem cells [38]. It appears feasible that transcriptomic analysis of gene panels in EUS FNA may be used for molecular stratification of pancreatic tumors as the basis to personalize therapeutic decisions, e.g. in a neoadjuvant treatment situation.

### mRNA/miRNA diagnostics is not suited for cyst fluid analysis of cystic tumors

Pancreatic cysts detected by imaging in asymptomatic patients may correspond to a variety of pathologies ranging from benign cysts (pseudocysts, serous cystic adenomas (SCA), true cysts) and premalignant or malignant cystic neoplasias (mucinous cystic neoplasms (MCNs), intraductal papillary mucinous neoplasms (IPMNs), solid pseudopapillary neoplasias (SPN), cystic pancreatic neuroendocrine neoplasias (cpNEN), serous cystadenocarcinomas). However, since up to 50-60% of the incidental pancreatic cysts detected by imaging show connections to pancreatic duct, they most likely represent IPMNs. One drawback of our study is that the complete set of data obtained from the combined mRNA/miRNA array did not allow to reliably identify cystic pancreatic neoplasias. Previous studies have reported that miRNA's, and in particular miR-21, miR155, miR-196a and miR-210, were able to differentiate malignant from non-malignant cystic lesion and in particular IPMNs ([22]; also see ([22] for an overview). Matthaei and coworkers [39] have used high-throughput miRNA analysis, followed by qRT-PCR in the same FFPE and pancreatic cyst fluid specimens, to develop a logistic regression model comprising 9 miRNAs, and correctly separated high risk from low risk pancreatic cysts with a sensitivity of 89% and a specificity of 100%. Wang et al. [40] used Next-Generation Sequencing (NGS) to study miRNA expression in a small number of EUS-FNAs from low-risk cysts (n = 6), high-risk cysts (n = 8), and PDACs (n = 3). Overall they found 13 up- and 2 downregulated miRNAs that, however, could not be all confirmed in validation experiments in the same and other studies (e.g.[22]). Frampton et al. in the most recently published trial reported that none of the miRNAs that were evaluated in EUS-FNA were able to separate benign non-mucinous (i.e. SCA and inflammatory/pseudocysts) from mucinous pancreatic cysts (i.e. MCN and IPMN) [22].

To summarize, our results and the results reported by other groups indicate that at present miRNA/mRNA diagnostics alone may be too variable to be reliably used in the clinical routine for the differentiation of pancreatic cysts. This may perhaps be resolved by introducing another genomic level in our multi-omic diagnostic platform. In a recent multi-center, retrospective study of 130 patients with resected pancreatic cystic neoplasms, cyst fluid was analyzed to identify mutations in genes known to be mutated in pancreatic cystic neoplasms (*BRAF*, *CDKN2A*, *CTNNB1*, *GNAS*, *KRAS*, *NRAS*, *PIK3CA*, *RNF43*, *SMAD4*, *TP53*, and *VHL*) [41]. With this combined analyses, the authors identified molecular markers and clinical features that classified cyst type with 90%-100% sensitivity and 92%-98% specificity. The molecular marker panel correctly identified 67 of the 74 patients who did not require surgery and thereby reduced the number of unnecessary operations by 91%.

It can thus be anticipated that a multi-omics diagnostic platforms as the one described here, combining mRNA, miRNA and mutational analyses with conventional diagnostic tests, such as cyst fluid CEA and cytology, will have the highest accuracy for the differentiation of the various types of benign and malignant pancreatic cysts.

### Limitations and outlook

Despite our promising results we recognize that our study has limitations. Our multi-omics platform comprises miRNAs from previously published studies selected for being specific for PDAC, and not for allowing to differentiate between other types of inflammatory, benign and malignant bilio-pancreatic masses. Our study is certainly underpowered concerning the various different types of bilio-pancreatic tumors, since these tumors are rare and FNA-material is difficult to obtain. This small sample size in some of the groups may have led to nonsignificant results due to a type II error. Furthermore, it may be argued that surgical FNAs may not be completely identical to the clinical situation, but we feel that this is a strength of our study, since histopathologically verified diagnoses for every single case are available as gold standard.

Our study provides evidence that a multi-omics platform assessing both, mRNA and miRNA biomarkers, is suitable for a molecular differentiation of suspect solid pancreatic masses. Further refinement of the selection of mRNA/miRNA markers will be done to allow the diagnosis of prognostic and predictive molecular PDAC subclasses such as those recently reported by Bailey and coworkers [14], that may have the potential to individualize the choice of neoadjuvant treatment. The limitations encountered in differentiating malignant and benign pancreatic cystic neoplasias was not completely unexpected based on the variable results reported by other groups. We are presently further developing the multiomics platform to allow additional mutational analyses of the genes that are known to differentiate pancreatic cystic neoplasias ([41], [42]) on the same platform in a single experimental step. We expect that this multi-omics platform will form an invaluable addition to the diagnostic workup of EUS-FNA biopsies of suspect solid and cystic pancreatic lesions.

## MATERIALS AND METHODS

### Tissue and biopsy samples

Since histological confirmation of diagnosis was an indispensable requirement for the validation of the performance of the diagnostic array, we focused on patients who received surgical resection of pancreaticobiliary masses for the purposes of this study. A total of 143 “surgical FNAB” (sFNAB) samples were performed from tumor masses immediately after resection using an 18G needle attached to a 20ml syringe, as previously described [11]. Biopsy material was expelled into 500 μl of RLT buffer (Qiagen) and the needle flushed again with the same volume of RLT buffer. Samples were stored at -80°C until processing. These sFNAB provided both accurate sampling of the tumor mass and subsequent histological evaluation of the very same tissue specimen.

Samples were provided by the surgery departments at the Ruprecht Karls University Heidelberg, Technical University Munich, and Philipps-University Marburg. Informed consent was obtained from all patients prior to using tissue or biopsy samples. The study was approved by the local ethics committees at the Universities of Marburg (Germany), Munich (Germany), and Heidelberg (Germany).

### RNA isolation, mixed TaqMan arrays and qRT-PCR analyses

Total RNA was isolated using the mirVana PARIS Kit (Thermo Fisher Scientific). We have previously developed protocols for simultaneous reverse transcription and pre-amplification of mRNA and miRNA targets as well as simultaneous analysis of mRNA and miRNA targets on a single TaqMan Array microfluidic card [23]. In short, 4 μL of RNA (20-200 ng) were reverse transcribed by combining a custom MicroRNA RT Primer Mix and a gene-specific pooled mRNA preamp Primer Mix (Thermo Fisher Scientific) using the TaqMan® microRNA Reverse Transcription Kit (Thermo Fisher Scientific). 5 μL of the reverse transcription product were used for pre-amplification using a custom gene-specific preamp mRNA/miRNA Primer Mix (Thermo Fisher Scientific) and the TaqMan® PreAmp Master Mix (Thermo Fisher Scientific). For qRT-PCR, the pre-amplified product was mixed with “TaqMan® Universal PCR Master Mix, No AmpErase UNG” (Thermo Fisher Scientific) and the appropriate amount of H_2_O, and subsequently loaded into the ports of the TaqMan array cards (2 loading ports to cover the total of 96 reaction chambers). RealTime PCR was performed on a 7900HT Fast Real-Time PCR System (with TaqMan® Array Block) (Thermo Fisher Scientific), using universal cycling conditions (95°C/10 min, then [95°C/15 sec, 60°C/60sec] for 40 cycles).

Raw C_t_ values were normalized separately for mRNA and miRNA genes, respectively. To this end, C_t_ values of the reference genes (RPLP0, PPIA, RPL37A, RPL30, RPS17 for mRNA genes; RNU44 and RNU48 for miRNAs) were averaged and subtracted from the C_t_ values of the individual marker genes. The resulting ΔC_t_ values were then used for biomarker selection and sample classification.

Details of the array composition are provided in Table 1; raw data can be accessed at http://www.staff.unimarburg.de/~buchhol3/DiagArray/

### Biomarker selection

In our experiments, we applied a biomarker selection process as a preprocessing step to the multivariate training of the classification model. The biomarkers that were discarded by the selection process were considered neither for the training nor for the application of the classification model [43, 44].

For the standalone evaluation of miRNA-markers, we exhaustively screened through all 2^9^-1 = 511 marker combinations, which was not applicable for the higher dimensional profiles of combined mRNA and miRNA markers.

For the combined analysis, the mRNA and miRNA markers were ranked according to the modified version of Threshold Number of Misclassification (TNoM) score [46]. The markers that achieved the highest scores on the training data were passed to the classification model. We conducted experiments with top lists of 1 up to 88 markers.

The basic version of the TNoM score calculates the accuracy of an univariate single threshold classifier for each individual marker [45]. For our experiments, we utilized a slightly modified version of the TNoM, which also takes into account the class-wise number of samples (TNoM_cw_) [46]. The TNoM_cw_ score can be seen as the mean sensitivity and specificity of a single threshold classifier.

### Classification model

We utilized a linear support vector machine (SVM) as multivariate classification model [47]. The decision boundary of a linear SVM can be seen as a linear hyperplane that separates feature space in two decision regions (e.g. PDAC/CP) (Figure 5iii). The SVM is designed as a large margin classifier, which maximizes the margin between the training samples and the decision boundary. The cost parameter of the SVM was fixed to a value of one. In our experiments, the input of the SVM is restricted to the biomarkers (mRNA/miRNA levels) selected by the biomarker selection process.

### Validation strategy

All marker selection experiments are conducted as 10×10 cross-validation experiments [48]. The 10×10 cross-validation procedure splits the available set of samples into 10 subsets (folds) of approximately equal size (Figure 2). The folds are repetitively grouped into independent training and validation sets. Nine folds are used for marker selection and training a classification model. The remaining fold is used for the validation of the used classifier. The procedure is repeated for each individual fold. In order to reduce sampling effects, the cross-validation is calculated for ten permutations (runs) of the overall dataset leading to 10×10 individual training/ test splits. The mean accuracy, sensitivity and specificity are reported. All experiments have been conducted in the TunePareto framework [49].

### Calculation of sensitivity, specificity and diagnostic accuracy

Histopathological evaluation of tumor tissue was used as the gold standard for diagnosis. For each diagnostic class, sensitivity of the molecular diagnostic procedure was calculated by dividing the number of true positive (TP) calls by the sum of the numbers of true positive and false negative (FN) calls: $\frac{\text{TP}}{\left(\text{TP }+\text{ FN}\right)}$

Specificity was defined as the number of true negative (TN) calls divided by the sum of the numbers of true negative and false positive (FP) calls: $\frac{\text{TN}}{\left(\text{TN }+\text{ FP}\right)}$

Diagnostic accuracy was defined as the total number of samples correctly assigned to their respective classes divided by the number of all classification calls made in the analysis.
